# Risk of Thrombosis in Patients Presenting with Myocardial Infarction with Nonobstructive Coronary Arteries (MINOCA)

**DOI:** 10.1055/s-0038-1645875

**Published:** 2018-05-03

**Authors:** Sivabaskari Pasupathy, Susan Rodgers, Rosanna Tavella, Simon McRae, John F. Beltrame

**Affiliations:** 1 Discipline of Medicine, University of Adelaide, Adelaide, South Australia, Australia; 2 Central Adelaide Local Health Network, Adelaide, South Australia, Australia; 3 Division of Hematology, SA Pathology, Adelaide, South Australia, Australia; 4 School of Pharmacy and Medical Sciences, University of South Australia, Adelaide, South Australia, Australia

**Keywords:** MINOCA, thrombophilia, coagulation factors, thrombosis

## Abstract

Patients presenting with myocardial infarction (MI) in the absence of obstructive coronary artery disease (CAD) is termed MI with nonobstructive coronary arteries (MINOCA). The underlying pathophysiology of MINOCA is multifactorial and in situ formation and subsequent spontaneous lysis of a coronary thrombus is often hypothesized as one of the mechanisms. The objective of this study is to determine whether MINOCA patients had a greater prothrombotic tendency in comparison to MI patients with obstructive CAD (MICAD). Prospectively, blood samples of 25 consecutive MINOCA patients (58 (interquartile range [IQR]: 48, 75) years, 48% women) and 25 age-/gender-matched MICAD patients (58 (IQR: 50, 66) years, 48% women) were obtained at 1 month after the initial presentation and overall thrombin generation potential and congenital/acquired thrombophilia states were assessed. As regard to results, overall thrombin generation parameters were similar ( *p* > 0.05) between the MINOCA and MICAD groups, highlighting similar endogenous thrombin potential (1,590 nM/min; IQR: 1,380, 2,000 vs. 1,750 nM/min; IQR: 1,500, 2,040, respectively). There were no significant differences between MINOCA and MICAD, respectively, in respect to the numbers of patients with congenital thrombophilia states including factor V Leiden (0 vs. 4%) and prothrombin gene mutation (8 vs. 4%), decreased antithrombin (8 vs. 0%), protein C (0 vs. 0%), and protein S (4 vs. 0%). None of the patients demonstrated presence of lupus anticoagulant and anticardiolipin antibodies. Although MINOCA patients revealed thrombotic characteristics that are similar to those with MICAD, the results from this study are inconclusive and a larger study with healthy control subjects is required to assess the risk of thrombosis in MINOCA.

## Introduction

Myocardial infarction (MI) with nonobstructive coronary arteries (MINOCA) is considered as a “working diagnosis” for patients presenting with a suspected myocardial infarct in the absence of obstructive coronary artery disease (CAD) on angiography. 1 The recent European Society of Cardiology (ESC) acute MI guidelines included MINOCA and highlighted it is imperative that the underlying cause is identified for each patient since this will influence subsequent therapy. 2 Multiple mechanisms have been proposed and one postulated mechanism for MINOCA is in situ thrombus formation with subsequent lysis, thereby resulting in a morphologically normal angiogram 3 but with the underlying causative prothrombotic state potentially predisposing to a further event. A recent systematic review has reported that as many as 14% of patients with MINOCA may have an abnormality detected on thrombophilia screening. 27 Congenital thrombophilia disorders detected in patients with MINOCA include factor V Leiden (FVL), prothrombin gene mutation (PGM), and proteins C and S deficiency. 27

The objective of this study is to compare the prothrombotic tendency of patients with MINOCA with that of MI patients with obstructive CAD (MICAD), by testing for known congenital and acquired thrombophilia conditions, markers of coagulation activation, and global coagulation by the thrombin generation assay. Our primary objective is to compare overall thrombin generation potential using thrombin generation test between MINOCA and MICAD. Secondary objectives are to compare congenital thrombophilia states, acquired thrombophilia states, and coagulation markers between these two groups.

## Methods

To achieve this objective, we employed a case–control study design recruiting age- and gender-matched patients with MINOCA and MICAD. Patients admitted for an acute MI at The Queen Elizabeth Hospital, Adelaide, Australia, were prospectively screened from May 2013 to March 2015 and were included if the following criteria were met: (1) fulfil the universal diagnostic criteria for an acute MI 4 based on troponin elevation with corroborative clinical criteria and (2) coronary angiography performed in the context of MI demonstrating MINOCA nonobstructive (<50% stenosis) coronaries or MICAD, obstructive (≥50% stenosis) coronaries. Patients were excluded from this study if they were on anticoagulant treatment, diagnosed with Takotsubo cardiomyopathy, and identified with noncardiac or chronic causes of troponin elevation such as heart failure, pulmonary disease, and chronic kidney disease.

Patients with confirmed MINOCA following coronary angiogram and sequential age- and gender-matched MICAD were consecutively approached and prospectively recruited into the study. All patients gave informed consent. The study was approved by the hospital human research ethics committee.

Blood sample was collected 4 weeks after the initial acute MI presentation, to avoid any influence from acutely administered drugs such as heparin or other anticoagulant agents, or activation of coagulation associated with the acute event. A minimal stasis using a 21-G needle into plastic 3.5-mL Vacuette tubes (Greiner Bio-One, Austria) containing buffered sodium citrate (final concentration: 0.105 mol/L), serum, and EDTA was used. Citrate plasma samples were processed within an hour of blood collection by a single centrifugation for 15 minutes at 2,200 g (4,000 rpm), with the top two-thirds of the plasma then removed, and stored in aliquots at −70°C.

Thrombin generation was measured using calibrated automated thrombin generation assay (CT, Thrombinoscope BV, Maastricht, The Netherlands) in a Fluoroscan Ascent fluorometer (Thermolab systems OY, Helsinki, Finland) using PPP reagent (5pM tissue factor, 4uM phospholipids, Thrombinoscope) as previously described by Rodgers et al. 5 MINOCA and matched MICAD samples were always tested in the same run, to avoid any effects due to variation between assays. In addition, two aliquots of quality control plasma samples and a commercial lyophilized plasma sample (HemosIL Calibration Plasma; Instrumentation Laboratory, Bedford, Massachusetts, United States) were also tested in the same run in each assay for validation. Assays were repeated if the quality control results were not in the desired range. The effect of thrombomodulin (TM) on thrombin generation was tested by the addition of rabbit lung TM (lot 140711; Sekisui, Stamford, Connecticut, United States), which was added into the reaction mixture at a final concentration of 0.35 unit/mL (5.89 nM). Readings from the fluorometer were automatically recorded and calculated using dedicated software (Thrombinoscope) that displays thrombin generation curves (time vs. generated thrombin) and calculates endogenous thrombin potential (ETP), peak thrombin, velocity index, lag time, and time to peak.

### Congenital Thrombophilia States

FVL and PGM were identified by primer extension genotype analysis using the Sequenom MassARRAY platform (Sequenom, San Diego, California, United States). The activities of antithrombin (AT, CV 4.7%) and protein C (PC, CV 2.8%) were assayed using a chromogenic substrate method, and free protein S antigen (PS, CV 4.7%) using latex immune-assay method on a STA-R analyser (Diagnostica Stago, France).

### Acquired Thrombophilia States

Lupus anticoagulant (CV 4.8%) was measured by a diluted Russell viper venom time assay (STA-Staclot DRVVT Screen, Stago). Anticardiolipin antibodies (CV 3.5%) were detected by a quantitative enzyme-linked immunosorbent assay (ELISA) kit (EUROIMMUN Medizinische Labordiagnostika AG, Germany) according to the manufacturer's instructions. Factor VIII coagulant activity (FVIII:C, CV 4.2%) was measured using a two-stage chromogenic assay (Biophen FVIII:C, Hyphen-Biophen, Neuville-sur-Oise, France), von Willebrand factor antigen (VWF:Ag, CV 15%) using latex immunoassay (STA-Liatest for VWF, Stago), and fibrinogen (CV 6%) using Clauss clotting method (STA-Fibrinogen, Stago) on a STA-R analyser (Diagnostica Stago).

### Coagulation Marker

D-dimer was measured using an immune-turbidimetric method (STA-Liatest D-DI, Stago) on a STA-R analyser (Diagnostica Stago).

Baseline characteristics, thrombin generation test variables (includes ETP, peak thrombin, lag time, and time to peak), and thrombophilia screen results were described in MINOCA patients in comparison to MICAD patients. Mean with range or medians with 25th and 75th percentiles were reported for continuous variables, and frequencies for categorical variables. Continuous variables were compared using either unpaired *t* -test with equal standard deviation or Mann–Whitney U-test. Categorical variables were compared using Fisher's exact test. A *p* -value of <0.05 was considered significant in all comparisons. Statistical analysis was performed using Graph Pad Prism software package for MAC OS X, version 6.0 (San Diego, California, United States).

## Results

Between May 2013 and March 2015, a total of 440 acute MI patients were admitted to The Queen Elizabeth Hospital. Of which 36 (8.2%) patients were identified as MINOCA. Twenty-five age- and gender-matched MINOCA (58 [48, 75], 48% women) and MICAD (58 [50, 66], 48% women) were recruited in each group for this study. Cardiovascular risk factors were similar between groups. The frequency of ST elevation myocardial infarction (STEMI) was higher in MICAD compared with that in MINOCA (84 vs. 12%, *p* < 0.05). In addition, MINOCA patients were less likely to receive secondary prevention treatment at discharge. Patients' baseline characteristics are listed in Table 1 . Blood samples were collected at a mean delay of 39 days from the acute presentation.

**Table 1:** Baseline characteristics

	MINOCA ( *n* = 25)	MICAD ( *n* = 25)	*p*
	Median (25th and 75th percentiles) or % ( *n* )			
Risk factors			
Age	58 (48, 75)	58 (50, 66)	0.09
Women	48% 12	48% 12	0.99
Hypertension	60% 15	52% 13	0.77
Hypercholesterolemia	64% 16	52% 13	0.56
Diabetes	24% 6	24% 6	0.75
Current smoker	24% 6	32% 8	0.75
Family history of CAD	28% 7	28% 7	0.99
STEMI	12% 3	84% 21	<0.001
Angiographic characteristics			
Normal coronaries (0% stenosis)	60% 15	0	–
Minor CAD (<50% stenosis)	40% 10	0	–
Obstructive CAD (>70% stenosis)	0	100% 25	–
PCI (stent insertion)	0	100% 25	–
Discharge medications			
Antiplatelet	64% 16	92% 23	<0.05
Second antiplatelet	8% 2	84% 21	<0.01
Statin	64% 16	96% 24	<0.05
ACE inhibitor	40% 10	84% 21	<0.05
ARB	12% 3	8% 2	0.99
Calcium channel blockers	44% 11	40% 10	0.99
Beta-blocker	20% 5	36% 9	034
Nitrates	20% 5	16% 4	0.99

Abbreviations: ACE, angiotensin converting enzyme; ARB, angiotensin receptor blocker; CAD, coronary artery disease; MICAD, myocardial infarction with coronary artery disease; MINOCA, myocardial infarction with nonobstructive coronary arteries; PCI, percutaneous coronary intervention; STEMI, ST elevation myocardial infarction.

Note: *p* < 0.05 considered statistically significant.

There was no statistically significant difference in ETP results between the MINOCA and MICAD groups (1,590 [1,380, 2,000] vs. 1,750 [1,500, 2,040]; Table 2 ). In addition, there were no statistically significant differences observed between MINOCA and MICAD in peak thrombin (300 [250, 380] vs. 340 [300, 390]), lag time (3 [2.9,4] vs. 3.3 [3, 3.9]), time to peak (6.7 [5.5, 7.4] vs. 5.9 [5.5,6.7]), and velocity index (107.1 [67.1, 147.8] vs. 128.5 [111.4, 144.9]) respectively, although there was a trend for higher values for ETP, peak, and velocity in the MICAD group. Addition of TM did not yield any statistically significant differences between the two groups either ( Table 2 ).

**Table 2:** Thrombin generation test parameters

	−TM			+TM		
	MINOCA	MICAD	*p*	MINOCA	MICAD	*p*
	Median (25th and 75th percentiles)			Median (25th and 75th percentiles)		
ETP (nM/min)	1,590	1,750	0.55	180	280	0.84
	(1,380, 2,000)	(1,500, 2,040)		(80, 390)	(150, 440)	
Peak thrombin (nM)	300	340	0.50	40	60	0.99
	(250, 380)	(300, 390)		(20, 90)	(30, 100)	
Lag time (min)	3.0	3.3	0.99	3.2	3.4	0.60
	(2.9, 4)	(3.0, 3.9)		(2.6, 4.6)	(2.8, 4.7)	
Time to peak (min)	6.7	5.9	0.54	5.7	6	0.99
	(5.5, 7.4)	(5.5, 6.7)		(5.1, 7.3)	(5.4, 6.7)	

Abbreviations: ETP, endogenous thrombin potential; MICAD, myocardial infarction with coronary artery disease; MINOCA, myocardial infarction with nonobstructive coronary arteries; TM, thrombomodulin.

Note: *p* < 0.05 considered significant.

Thrombophilia screening results are shown in Table 3 . Neither mean levels of the congenital inhibitors of coagulation (AT, protein C, and protein S) nor the incidence of patients with abnormally low test results differed significantly between MINOCA and MICAD patients. The prevalence of patients with the FVL or PGM mutations was low in both the MINOCA and MICAD groups (0 vs. 4%, 8 vs. 4%) and did not significantly differ ( *p* > 0.05). Antiphospholipid antibodies (lupus anticoagulant and anticardiolipin antibodies) were not found in any of the patients from both groups. Mean levels of F VIII:C, VWF:Ag, and fibrinogen were not significantly different between MINOCA and MICAD patients, and the incidence of patients with results above the normal range did not differ significantly. The proportion of patients with elevated D-dimer level (44 vs. 36%, *p* > 0.05) was similar in both groups, and mean levels did not differ significantly.

**Table 3:** Incidence and expression of thrombophilia states

Tests	MINOCA	MICAD	*p*
	Median (25th and 75th percentiles) or % ( *n* )			
Congenital thrombophilia			
Antithrombin III units	101 (72, 134)	100 (82, 122)	0.86
Antithrombin deficiency	8% 2	0	0.22
Protein C units	121 (65, 293)	116 (78, 189)	0.74
Protein C deficiency	0	0	–
Protein S units	111 (49, 142)	104 (67, 150)	0.18
Protein S deficiency	4% 1	0	0.47
Factor V Leiden	0	4% 1	–
Prothrombin gene mutation	8% 2	4% 1	0.59
Acquired thrombophilia			
Anticardiolipin antibody (GPL ^a^ units)	1.5 (1, 4)	2.3 (1, 18)	0.38
Antiphospholipid antibody syndrome	0	0	–
Presence of lupus anticoagulant	0	0	–
Factor VIII (IU/dL)	168 (61, 301)	159 (78, 229)	0.72
Above normal (>180)	44% 11	36% 9	0.42
von Willebrand factor antigen (IU/dL)	151 (60, 299)	147 (57, 274)	0.88
Above normal (>240)	12% 3	4% 1	0.17
Coagulation markers			
Fibrinogen units	3.7 (2.2, 5.4)	3.8 (2.1, 5.7)	0.53
Above normal (>4.0)	44% 11	40% 10	0.58
D-dimer units	0.6 (0.1, 1.9)	0.4 (0.2, 0.9)	0.47
Above normal (>0.5)	44% 11	36% 9	0.41

Abbreviations: dRVVT, dilute Russell's viper venom time; MICAD, myocardial infarction with coronary artery disease; MINOCA, myocardial infarction with nonobstructive coronary arteries.

Note: Data expressed as either median (25th and 75th percentiles) or frequencies; *p* < 0.05 considered significant.

a GPL denotes IgG isotype.

## Discussion

Underlying thrombophilia resulting in an increased tendency to intravascular thrombosis have been postulated as one of the possible causes of MINOCA in previous studies and reviews. 3 6 This study is the first of its kind to examine the thrombin generation in this patient group. It demonstrated similar thrombin generation activity in MINOCA patients to a matched MICAD population, along with no difference in the results of testing for recognized congenital or acquired thrombophilia states, and similar D-dimer results.

Maiwald et al 7 utilized a similar method to compare the thrombin generation characteristics between MINOCA, MICAD, and healthy controls. The study demonstrated thrombin generation parameters were similar between MINOCA and MICAD and higher compared with healthy controls. Although the study was hampered by small sample size, it warranted MINOCA and MICAD thrombin generation aspects. Similar to Maiwald et al's study, present study also demonstrated similar overall thrombin generation parameters (specific features including ETP, peak thrombin, lag time, time-to-peak, and velocity index) between MINOCA and MICAD. Potential explanations for this finding include the possibility that MINOCA patient's small plaque rupture, not detectable by angiography, may initiate localized thrombosis leading to arterial obstruction. Such vessel wall abnormalities would not influence thrombin generation results. The potential role of such a mechanism was highlighted by Reynolds et al, 8 who demonstrated plaque rupture in 16 of 42 female patients (38%) undergoing intravascular ultrasound (IVUS) following MINOCA presentation. MINOCA patients may therefore potentially benefit from IVUS to screen for plaque/clot rupture. In addition, the role of spasm in these patients could not be tested, but spasm may also initiate a partial stenosis leading to secondary thrombosis or provoke plaque rupture initiating the coagulation cascade.

Evidence of the association between deficiencies of AT, PC, or PS and arterial thrombosis is limited to case reports and small studies that are generally hampered by low prevalence of these thrombophilia states similar to the current study. None of the patients demonstrated AT deficiency in studies investigated by Rallidis et al 9 among 70 acute MI patients before the age of 36 years and by Dacosta et al 10 among 75 acute MI patients before the age of 45 years. Da Costa et al 6 11 presented only 1 patient with protein C or S deficiency in two studies with 73 and 78 MINOCA patients.

The presence of FVL is the most common risk factor for venous thrombosis and has often been associated with MINOCA. Da Costa et al, 6 Van de Water et al 12 and Mansourati et al 13 demonstrated around 10% of MINCOA patients with FVL. However, none of the MINOCA patients in the current study exhibited this gene mutation. Mansourati et al 13 demonstrated 12% of 107 MINOCA patients with FVL in comparison to 4.5% of 244 with MICAD. Rosendaal et al 14 demonstrated FVL as a risk factor for acute MI in young women (<44 years). Van de Water et al 12 showed increased frequency of PGM in young MINOCA patients compared with young MICAD. It is important to note that the patient cohorts in these studies are primarily younger compared with the present study. Among the studies reporting inherited coagulation disorders in MINOCA, Van de Water et al 12 demonstrated that MINOCA patients younger than 50 years are more likely to express either FVL or PGM compared with patients older than 50 years (20 vs. 6%, *p* < 0.05), whereas the mean age of our MINOCA group was 58 (range: 18–87). Ethnicity also plays a crucial role in inherited thrombophilic states. Caucasians are more likely to exhibit FVL compared with any other races outside Europe. 15 Our data did not clearly identify the presence of a congenital thrombophilia state as a common risk factor in unselected patients with MINOCA, although previous studies suggest that young patients may be at high risk of developing acute MI in the presence of a congenital thrombophilia state, particularly when classical risk factors such as smoking are present.

Anticardiolipin antibodies were shown to be a rare independent risk factor for MI and recurrent events. The role of anticardiolipin antibodies in the pathophysiology of arterial vascular thrombotic events is well established. 16 17 Segev et al 18 demonstrated the incidence of anticardiolipin antibodies in 18% of 85 STEMI patients younger than 50 years in whom percutaneous coronary intervention was performed. Davies et al 19 presented a case series with five MINOCA patients who were found to have lupus anticoagulant and/or anticardiolipin antibodies and suggested MINOCA patients may benefit from screening for antiphospholipid antibodies. Dacosta et al 20 also showed a rare case of a MINOCA patient in whom thrombosis was caused by antiphospholipid syndrome. Although, the present study did not identify any patients with antiphospholipid syndrome, screening may benefit some young MINOCA patients.

Elevated FVIII is found in 11% of general adult population 21 and elevated levels are more common in women, patients with blood groups other than O, patients with high body mass index, diabetics, and in clinical conditions like chronic inflammation. In many cases, there was concomitant elevation in both FVIII and VWF:Ag. 22 Elevation in acute phases may not return to baseline for several months. There are reported cases of acute coronary syndrome associated with elevated FVIII:C with no other cardiovascular risk factors or significant atheromatous disease. 23 24 The relationship between FVIII:C and venous thromboembolism is well documented, 25 but the role in arterial thrombosis is not clear as yet. Significant elevation of FVIII:C in both groups was noted in the present study; whether this elevation was associated with an acute phase response could not be clearly ruled out, and later testing may have been of benefit.

Elevated plasma fibrinogen levels, whatever their origin, may cause a hypercoagulative state that could influence the degree and duration of thrombus formation at the time of coronary injury. Previous reports suggest that fibrinogen is an independent risk factor for premature AMI. 26 The present study showed slightly raised levels of fibrinogen in both groups, which could be due to the MI or an underlying vascular disease than as a cause of MI.

D-Dimer, an expression of on-going thrombus formation and lysis, is a marker of substantial incremental value for the early diagnosis of acute coronary syndromes. It adds independent information to the traditional assessment for acute MI. Although there are no previous studies comparing the elevated D-dimer in MINOCA with that in MICAD, because of the wide overlap between the groups, increased D-dimer values are of limited relevance above and beyond other risk factors. Nonetheless, it demonstrates increased fibrinolytic activity 4 weeks' post-MI in both groups.

## Limitations

The results from this study should be interpreted in the context of several potential limitations. Thrombin generation assay does not measure the cell components of coagulation, thus giving a partial view of the hemostatic system. We also did not examine thrombin generation using a lower trigger concentration of tissue factor (1pM). The relatively small sample size may also explain the negative findings, particularly regarding the rarer inherited deficiency states. A sample size calculation for congenital thrombophilia states based on Dacosta et al's findings revealed that to test the proportion difference of 7.5 and 18.2%, at 80% power and 5% significance, 171 patients in each group would be required. It will also be beneficial to compare the MINOCA and MICAD cohort to age- and gender-matched healthy cohort in whom prior MI or CAD is not documented. In addition, the higher incidence of non-STEMI in the MINOCA group is also a limitation.

## Conclusion

In summary, overall thrombin generation potential, congenital thrombophilia states, acquired thrombophilia states, and coagulation markers in this study were not different between MINOCA and MICAD patients, suggesting that despite the difference in coronary artery anatomy of the disease progression, acute MI patients generate thrombin in a similar manner in response to local stimuli. Although the role of an abnormal prothrombotic tendency is often hypothesized to be causative in the setting of MINOCA, whether testing for such an underlying condition helps in the clinical management of MINOCA is questionable. From our findings, the testing for hereditary thrombophilia would not alter the clinical management of patients; however, it could provide important mechanistic insights only in a minority of patients.
